# Elements That Contribute to Healthy Building Design

**DOI:** 10.1289/ehp.8988

**Published:** 2007-01-25

**Authors:** Vivian Loftness, Bert Hakkinen, Olaf Adan, Aino Nevalainen

**Affiliations:** 1 Carnegie Mellon University, School of Architecture, Pittsburgh, Pennsylvania, USA; 2 Gradient Corporation, Cambridge, Massachusetts, USA; 3 TNO Built Environment and Geosciences, Delft, the Netherlands; 4 National Public Health Institute, Department of Environmental Health, Kuopio, Finland

**Keywords:** consumer products, dampness, emissions, fungal resistance, healthy buildings, indoor air, sustainable development, ventilation

## Abstract

**Background:**

The elements that contribute to a healthy building are multifactorial and can be discussed from different perspectives.

**Objectives:**

We present three viewpoints of designing a healthy building: the importance of sustainable development, the role of occupants for ensuring indoor air quality, and ongoing developments related to indoor finishes with low chemical emissions and good fungal resistance.

**Discussion:**

Sustainable design rediscovers the social, environmental, and technical values of pedestrian and mixed-use communities, using existing infrastructures including “main streets” and small-town planning principles and recapturing indoor–outdoor relationships. This type of design introduces nonpolluting materials and assemblies with lower energy requirements and higher durability and recyclability. Building occupants play a major role in maintaining healthy indoor environments, especially in residences. Contributors to indoor air quality include cleaning habits and other behaviors; consumer products, furnishings, and appliances purchases, as well as where and how the occupants use them. Certification of consumer products and building materials as low-emitting products is a primary control measure for achieving good indoor air quality. Key products in this respect are office furniture, flooring, paints and coatings, adhesives and sealants, wall coverings, wood products, textiles, insulation, and cleaning products. Finishing materials play a major role in the quality of indoor air as related to moisture retention and mold growth.

**Conclusions:**

Sustainable design emphasizes the needs of infrastructure, lower energy consumption, durability, and recyclability. To ensure good indoor air quality, the product development for household use should aim to reduce material susceptibility to contaminants such as mold and should adopt consumer-oriented product labeling.

A healthy building is based on the successful fulfillment of many requirements. For each building, sound design and construction are necessary for its technical functioning and mechanical stability and for the basic safety of its occupants. However, this is not sufficient to ensure indoor environmental quality (IEQ) for its occupants. There are a number of other factors that affect the occupants’ well-being either directly or indirectly. Among such factors are heating, ventilation and air conditioning, and activities of the occupants, including the use of office equipment or household activities such as cooking, cleaning, or applying pesticides. The risk assessment of indoor contaminants and the effectiveness of interventions are challenges faced globally because of vast differences in the types of residences and their climates as well as the many types of household products, furniture, appliances, and so on, that are available to consumers today. Examples of these diverse challenges have been demonstrated in the book *The Material World* that provides detailed, thought-provoking visual and written portraits of “statistically average” families and their households in 30 nations around the world (Menzel 1994).

Indoor air pollution is not a new problem, although only recently has it become a matter of public concern. As early as the 18th century, hygienists had identified the consequences of inadequate ventilation in the indoor environment. Systematic research activities emerged soon after World War II, in some respects reversed by energy conservation measures introduced in housings after the oil crisis in the early 1970s. Since then, the complexity and the health relevance of the indoor environmental problem have become increasingly apparent (European Commission 2005a, 2005b).

Failures to control indoor air risks have huge economic consequences in the form of health care costs, lost working days, and personal costs to individuals (Mendell et al. 2002). Consequently, investments in developments that pursue enhanced human health and well-being through healthier indoor environments should not be seen as business nuisances but should be weighed against the benefits gained. Because factors contributing to building health are complex, with connections to many essential fields, we do not attempt to cover all aspects but present three essential ideas: sustainable development of buildings and communities, the effect of occupants on the indoor environment, and recent developments in creating healthier products and building materials with a focus on moisture and mold control. These three areas are important because they address the most current issues in building design: sustainability (in terms both of natural resources and of the lifetime of the building); individual behaviors and how they affect their indoor environments; and the newest trends in building materials that can promote healthier indoor environments.

## Environmental Sustainability Contributes to Health, Productivity, and Quality of Life

Sustainable design is a collective process whereby the built environment achieves ecologic balance in new and retrofit construction toward the long-term viability and humanization of architecture. In an environmental context, this process merges the natural, minimum resource-conditioning solutions of the past (daylight, solar heat, natural ventilation) with the innovative technologies of the present into an integrated “intelligent” system that supports individual control to achieve environmental quality with resource consciousness. Sustainable design rediscovers the social, environmental, and technical values of pedestrian, mixed-use communities, fully using existing infrastructures, including “main streets” and small-town planning principles and recapturing indoor–outdoor relationships. It attempts to avoid the thinning out of land use and the dislocated placement of buildings and functions caused by single-use zoning. Sustainable design introduces benign, nonpolluting materials having lower operating energy requirements and higher durability and recyclability. Finally, sustainable design offers architecture of long-term value through modifiable building systems through life-cycle instead of least-cost investments and through timeless delight and craftsmanship (Loftness et al. 2005).

The importance of proving that sustainable design and engineering improves health, productivity, and quality of life has never been more important. To this end, the Center for Building Performance at Carnegie Mellon University in collaboration with the Advanced Building Systems Integration Consortium (ABSIC) from 2000 to the present have been developing a building investment decision support tool—BIDS (Carnegie Mellon, Pittsburgh, PA). This cost–benefit tool presents the life-cycle data of over 200 case studies—laboratory, field, and simulation studies that reveal the substantial environmental benefits of a range of advanced and innovative building systems. The health benefits of high-performance buildings designed to deliver high-quality air, thermal control, light, ergonomics, privacy, and interaction as well as access to the natural environment were analyzed (Center for Building Performance and Diagnostics/Advanced Building Systems Integration Consortium 2005). The following components were included:

- healthy, sustainable air;
- healthy, sustainable thermal control;
- healthy, sustainable light;
- workplace ergonomics and environmental quality;
- access to the natural environment; and
- land use and transportation.

### Healthy, sustainable air

This component depends on commitments to improve the quality and quantity of outside air, maximize natural ventilation with mixed-mode heating, ventilating, and air-conditioning (HVAC) systems, and separate ventilation air from thermal conditioning, provide task air and individual control, and improve pollution source control and filtration. International case studies have demonstrated that high-performance ventilation strategies reduce respiratory illness 9–20% and increase individual productivity between 0.48 and 11%, with a small energy cost for increasing outside air rates with heat recovery, or 25–50% energy savings for natural ventilation and mixed-mode conditioning (e.g., Fisk and Rosenfeld 1997; Kroeling et al. 1988).

### Healthy, sustainable thermal control

This second component depends on commitments to separate ventilation air from thermal conditioning, design for dynamic thermal zone size, provide individual thermal controls (e.g., underfloor air), design for building load balancing and radiant comfort, and engineer prototyped, robust systems. International case studies demonstrate that providing individual temperature control for each worker increases individual productivity by 0.2–3% and reduces sick building syndrome (SBS) symptoms and absenteeism, while saving 25% of conditioning energy (e.g., Wyon 1996).

### Healthy, sustainable light

The third component can be achieved by maximizing the use of daylight without glare, selecting the highest quality lighting fixtures, separating task and ambient light, and designing plug-and-play lighting with dynamic lighting zones. Case studies demonstrate that improved lighting design increases individual productivity between 0.7 and 23%, reduces headaches and SBS symptoms by 10–25%, while reducing annual energy loads by 27–88% (Heschong et al. 2002*).*

### Workplace ergonomics and environmental quality

Improving this fourth component has, as its goals, the well-being and efficiency of individual workers with energy-efficient technologies; optimal lighting, temperature, and placement of furniture; and healthy interior materials. Sustainable design depends on the use of materials that support healthy environments while reducing transportation energies that carry secondary health concerns. Material selection is critical to thermal performance, air quality and outgassing, toxicity in fires, cancer-causing fibers, and mold, all which affect respiratory and digestive systems, eyes, and skin (Dainoff 1990*).*

### Access to the natural environment

The fifth component is achieved by providing individual access to nature by maximizing the use of daylight without glare, maximizing the use of natural ventilation with mixed-mode HVAC, and designing for passive solar heating and cooling. Access to the natural environment may increase individual productivity between 0.4 and 18% and reduce absenteeism, SBS, and recovery time while saving even 40% of lighting energy (Center for Building Performance and Diagnostics/Advanced Building Systems Integration Consortium 2005).

### Land use and transportation

This last component can be improved by commitments to designing mixed-use communities, allowing for multigenerational mobility with mixed-mode transportation, and preserving and celebrating natural landscapes. For land use, walkable neighborhoods may contribute to prevention of obesity (Srinivasan et al. 2003). Cool roofs and cool community developments with increases in landscaped surfaces and tree canopies demonstrated reductions in annual cooling loads by 10%, peak cooling by 5%, as well as benefits for carbon sequestration, storm runoff management, and a 6–8% reduction in smog that could potentially reduce respiratory illnesses (Rosenfeld and Romm 1997).

## Quantifying the Value of the Built Environment to Health

**Figure 1:**
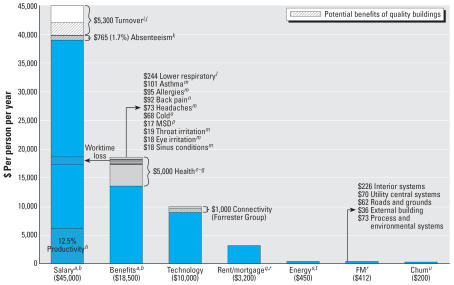
Improving the quality of the built environment will reduce the life cycle costs of business. Monetary amounts are in U.S. dollars per year. MSD, musculoskeletal disorders. Forrrester Group is part of Forrester Research (Cambridge, MA). Data from ***a***U.S. Department of Labor (DOL) (2004a); ***b***U.S. DOL (2004b); ***c***U.S. DOL (2002); ***d***Kaiser Family Foundation and Health Research and Educational Trust (2003); ***e***Towers Perrin HR Services (2003); ***f***U.S. Chamber of Commerce (2003); ***g***Deloitte & Touche (2003); ***h***Leaman (2001); ***i***U.S. DOL (2003b); ***j***Fitz-Enz (2000); ***k***U.S. DOL (2003a); ***l***Birnbaum et al. (2003); ***m***U.S. EPA (1998); ***n***Guo et al. (1999); ***o***Fendrick et al. (2003); ***p***Silverstein et al. (2000); ***q***General Services Administration (2003); ***r***International Facility Management Association (IFMA) (2002); ***s***U.S. DOE (1998); ***t***U.S. Department of Energy (DOE) (2004); ***u***IFMA (2001).

It is imperative to incorporate the full life-cycle costs of a poor-quality built environment, from materials to systems to land use and transportation. Based on health insurance costs reported in five references by independent nonprofit organizations, human resource research firms, and the U.S. government, the average employer cost for health insurance was approximately US$5,000 per employee per year in 2003 (Figure 1). Some health conditions and illnesses have been linked to the quality of the indoor environment, including colds, headaches, respiratory illnesses, musculoskeletal disorders, back pain, and symptoms of SBS. These are presented in Figure 1 with references.

Suboptimal indoor environments can lead to a variety of adverse health effects that result directly in increased physician visits and medical treatment. This leads to increases in health insurance costs, both for institutions and for individuals. Improvements in indoor environments, such as increased ventilation rates, better ergonomics and lighting, and improved heating and cooling methods, would reduce many of the adverse symptoms and illnesses described above.

**Figure 2:**
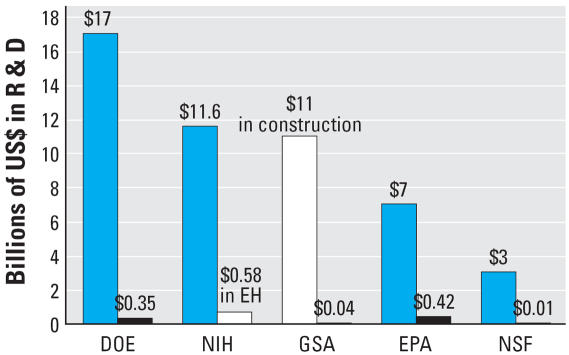
U.S. government investments (US$) in research to achieve healthy indoor environments (Office of Management and Budget 1998). Abbreviations: DOE, Department of Energy; EH, environmental health; EPA, U.S. Environmental Protection Agency; GSA, General Services Administration; NIH, National Institutes Health; NSF, National Science Foundation. Blue bars, total U.S. federal research funding; black bars, U.S. built environment research funding; GSA white bar, total construction dollars, not total research dollars; NIH white bar, environmental health research funding but not directly built environment research funding.

Human health in the built environment is one of the most critically needed research efforts, requiring both extensive experimental and field research. Controlled laboratory experiments need to be carried out simultaneously with experiments in actual buildings to map chains of consequence and to identify possible building-related causes for the rise in respiratory problems, fatigue, stress, depression and other health-related declines in the quality of life. Yet there is remarkably little federal investment in defining and valuing healthy buildings and communities (Figure 2).

The opportunity to substantially improve the health of building and community residents through investments in higher quality materials, systems, and land-use planning is significant. The catalyst for these investments must be research and subsequent policy based on the combined expertise of the health research community and the sustainable design and engineering disciplines that we hold responsible for our built environment.

## Human Influence on Healthy Indoor Air

Humans have a major role in maintaining the quality of the indoor environments in which they live. Lifestyles that affect IEQ include the following:

- Personal cleaning habits. Examples include frequency of vacuuming and washing of bed linen and towels.
- Other personal behavior such as whether kitchen or bathroom fans are commonly used and whether windows are opened to increase air circulation if certain consumer products are used.
- The types of consumer products that are purchased and where and how the consumer and other occupants of the residence use them.
- Decisions about the types of house or apartment furnishings that are purchased, for example, the presence of carpets and curtains in various rooms, and remodeling choices.
- Decisions about the types of appliances that are purchased, for example, a central air cleaning system or a high-efficiency vacuum cleaner.
- Personal cleaning habits.

Examples of the sources of indoor pollutants such as lead, pesticides, polycyclic aromatic hydrocarbons (PAHs), allergens, and volatile organic compounds (VOCs) include consumer products, the dust present in carpets and furniture, household pets, or pollutants entering the house from outside air. The accumulation of dust, dust mites, and tracked-in soil in old carpets, sofas, and mattresses appears to be a major source of exposure to lead, pesticides, allergens, PAHs, and VOCs and can be affected by cleaning habits such as the frequency of vacuuming and the washing of bed linen and towels (Roberts and Dickey 1995).

### Other personal behaviors in indoor environments

Personal behaviors such as opening windows and using exhaust fans can have significant impacts on reducing exposures from activities such as paint stripping (Riley et al. 2000). Window-opening behaviors can have a strong effect on a home’s air change rate; thus, this factor should be incorporated into exposure analyses when estimating human exposure to indoor air pollutants (Howard-Reed et al. 2002). Behaviors related to heating and cooling the building can also affect the air-exchange rate and the prevalence of microbial and chemical contaminants (Flannigan and Miller 2001). Common household water-use activities such as showering, clotheswashing, handwashing, bathing, dishwashing, and indirect shower exposure can increase indoor chemical exposures by inhalation of vaporized or aerosolized chemicals and by inadvertent ingestion of water. For example, some of the greatest increases in systemic exposure to trihalomethanes (THM) have been associated with showering (direct and indirect), bathing, and hand dishwashing (McKone 2005; Nuckols et al. 2005). Activities such as cooking, arts and crafts, cleaning floors, and painting can contribute to short-term increases in indoor VOC levels. Diminished VOC levels were achieved by turning on the air-conditioning system (Clobes et al. 1992). Activities shown to generate considerable amounts of indoor particulate matter include cooking, smoking, cleaning, sources such as cigarette side-stream smoke, pure wax candles, scented candles, a vacuum cleaner, an air-freshener spray, a flat iron (with or without steam) on a cotton sheet, electric radiators, and electric and gas stoves (Afshari et al. 2005).

A study by Ferro et al. (2004) of the personal, indoor, and outdoor particulate matter (PM) concentrations for a variety of prescribed human activities found that the activities that resulted in the highest exposures to PM with aerodynamic diameters ≥ 2.5 μm (PM_2.5_), ≥5 μm (PM_5_), and ≥ 10 μm (PM_10_) were those such as dry dusting, folding clothes and blankets, and making beds. Such activities disturbed dust reservoirs on furniture and textiles. The vigor of activity and type of flooring were also important factors for dust resuspension. The findings demonstrate that a wide variety of indoor human resuspension activities increases human exposure to PM and contributes to the “personal cloud” effect (Ferro et al. 2004).

### Consumer products and their use in residences

Various household products can be used alone or together with other products for cleaning, cosmetics, or a variety of other purposes. Consumer studies have found that there can be large intra- as well as interindividual variation in the frequency, duration, and amount of use of products such as dishwashing detergents, pesticides, cleaning products, and hair-styling products (Weegels and van Veen 2001). Common household activities can raise exposures to volatile organic chemicals (VOCs) up to a factor of 100 compared with exposures during the sleep period and far above the highest observed outdoor concentrations. Major associations of consumer products with particular indoor chemical exposures include deodorizers and the level of *p*-dichlorobenzene, dishwasher and laundry detergents and the level of chloroform, smoking and the levels of benzene and styrene, and painting and using paint remover and the levels of *n*-decane and *n*-undecane (Wallace et al. 1989).

Moreover, combinations of consumer products, or a mix of consumer products with outdoor air, can produce respiratory tract irritants. Cleaning agents and air fresheners can contain chemicals that react with other air contaminants to yield potentially harmful secondary products. For example, terpenes from consumer products can react with ozone in indoor air to generate secondary pollutants (Clausen et al. 2001; Nazaroff and Weschler 2004).

### Home furnishings and decorating

Decisions about home furnishings and decoration, such as the types of furniture purchased, the presence of carpets and curtains in various rooms, and remodeling choices, can also affect indoor contaminant exposures. For example, the remodeling of a residence and the adoption of energy conservation methods can reduce ventilation and increase relative humidity. The changes in these factors could increase the levels of dust, dust mites, molds, VOCs, and other indoor air pollutants (Roberts and Dickey 1995).

### Household appliances

Decisions about the types of appliances that are purchased can be driven partly by personal cleaning habits, for example, how clean the residence is kept. Further, using air-conditioning while sleeping can lead to a considerable build-up in the room of carbon dioxide (CO_2_) from all types of air-conditioning systems. These CO_2_ levels were substantially higher than the levels in naturally ventilated bedrooms. A survey was conducted to investigate whether the occupants exhibited symptoms of SBS while sleeping in air-conditioned as well as naturally ventilated bedrooms. Almost all occupants who used air-conditioning while sleeping exhibited one or more SBS symptoms and usually displayed more SBS symptoms after using air-conditioning than when they used natural ventilation. The survey also revealed that the frequency and duration of using air-conditioning has an important impact on the exhibition of the SBS symptoms (Wong and Huang 2004).

## Ongoing Developments in Controlling Emissions from Products and Building Materials

Today, more consumer products and building materials are being studied and certified as low chemical-emitting products and materials to serve as primary control measures for achieving good indoor air quality. Key products identified by the U.S. Environmental Protection Agency (EPA) as sources of indoor air pollution are office furniture, flooring, paints and coatings, adhesives and sealants, wall coverings, office equipment, wood products, textiles, insulation, and cleaning products. Product emission testing protocols have been designed to help ensure that the test results can be translated into real-world product usage scenarios.

The American Society for Testing Materials (ASTM) has established guidelines for measuring chemical emissions using environmental chambers. ASTM D5116-97 (ASTM 2007a) and D6670-01 (ASTM 2007b) are the foundation for some product-specific test protocols. One testing laboratory, the Greenguard Environmental Institute (GEI) in Atlanta, Georgia, has established performance-based standards to label goods with low chemical and particle emissions for use indoors, primarily building materials, interior furnishings, furniture, cleaning and maintenance products, electronic equipment, and personal care products. The standards of GEI establish certification procedures, including test methods, allowable emissions levels, product sample collection and handling, testing type and frequency, and program application processes and acceptance (GEI 2005). The Carpet and Rug Institute’s “Green Label” Testing Program for Carpets and Vacuum Cleaners in Dalton, Georgia, is another example of testing and certification of low-emitting products (Carpet and Rug Institute 2005).

“Smart” construction materials and coatings are being developed through a test program for innovative construction materials, with the goal of decreasing indoor air pollution. One example is the PICADA (Photocatalytic Innovative Coverings Applications for De-Pollution Assessment) project, involving a European consortium of private enterprises, research institutions, and the European Commission’s Joint Research Centre. The “smart” construction materials (plaster, mortar, architectural concrete) and coatings contain titanium dioxide (TiO_2_). Nitrogen oxide (NO_x_) gases and organic compounds diffuse through the porous surface of the materials and coatings and stick to the TiO_2_ nanoparticles. Absorption of ultraviolet light by the TiO_2_ leads to its photoactivation and the subsequent degradation of the pollutants adsorbed onto the particles. The acidic products created by this process are washed away by rain and/or neutralized by alkaline calcium carbonate contained in the materials. Such new construction materials could help to reduce levels of NO_x_ gases that cause respiratory problems and trigger smog production, and of other toxic substances such as benzene.

Tests with photocatalytic materials under field conditions have shown that outdoor air quality can be significantly improved. For example, up to 60% reduction in the concentration of NO_x_ at street level was detected after 7,000 m^2^ of road surface in Milan, Italy, were covered with a photocatalytic cementlike material. Such new construction materials and coatings could play a major role in helping meet the European Union (EU) target of reducing NO_x_ levels to < 21 ppb/year by 2010. Although EU researchers have focused on the development of these types of materials for outdoor applications, future work is planned to determine whether these products can also be used as depolluting building materials and coatings in indoor environments (PICADA 2005).

## Fungal Resistance of Construction Materials and Finishes

Dampness, moisture, and mold problems in buildings are a major factor affecting the quality of indoor air worldwide [Institute of Medicine (IOM) 2004]. These phenomena have a well-documented link to health effects such as respiratory symptoms and asthma (Bornehag et al. 2001, 2004; IOM 2004; Peat et al. 1998). Various signs of dampness or moisture damage are common in modern buildings (Nevalainen et al. 1998), and the prevalence of observations of mold varies from 1.5–20% (Bornehag et al. 2005; Anonymous 1993).

Dampness and mold are complex problems both from the point of view of building construction and human health. Although fungal spores are present everywhere, it is when dampness and moisture are uncontrolled that fungi grow and thus develop into visible mold. Use of fungicides or disinfection products do not solve the problem and may even be an additional load to indoor chemical exposures. Moisture control may be difficult to manage in existing buildings, and therefore any delay in the development of actual mold damage allows time for drying of the moistened materials. It is evident that the materials of a healthy building should be sturdy and resistant to microbial growth. It is also evident that both dissemination of information and access to training about the risks of dampness and mold are necessary for control of the problem. Training should be directed to professionals in building design and construction as well as in building maintenance, management, and renovation. Furthermore, the general public, as the users and occupants of buildings, plays an important role in prevention and control of these problems. Therefore, their awareness of the risks of dampness and interventions to control it is critical.

Adan (1994) found that the finishing materials on buildings play a pivotal role in mold growth and the quality of the indoor environment. Effects are most pronounced in places with highly transient moisture loads such as bathrooms. Regardless of insulation levels and even with high ventilation rates, moistening of surfaces cannot be avoided. Moisture retention in the finish may cause sustained high surface humidity, even when the indoor air is dry. This explains why, in modern highly insulated dwellings in cold and temperate maritime climates, mold risk is primarily a matter of material properties. Considering the industrial trend toward ecofriendlier products, which is generally accompanied by an increase in constituent biodegradability, the situation is growing worse.

Therefore, a sustained strategy of indoor fungal growth control must consider the pivotal role of finishing products. Two major developments are promising:

- Research and development is under way in the supply industry, with the goal of reduced material susceptibility. This initiative is driven primarily by environmental legislation and concerns biocides in particular.
- Performance requirements in building codes and/or consumer-oriented product labeling are being considered for finishes. The finishing materials very often are a designer’s or consumer’s choice. Labeling can make the end-user conscious of the consequences.

### Reducing biosusceptibility

Presently, sufficient resistance of materials to microbial attack requires addition of biocides, with paints being the main application area. There are two major technical limitations in terms of release and environmental impact.

First, the activity period of the biocide is usually much shorter (maximum 1–2 years) than the desired service life of the finish, leading to early replacement. Biocides tend to leach out quickly in the early stages of the coating’s lifespan, thereby decreasing the amount of active material available for the longer term. Raising initial biocide concentrations tries to counter this effect. Biocides must be sufficiently mobile to find their way to the surface. Consequently, biocides are inherently sensitive to leaching, especially when the surface is in direct contact with water.

To prolong the effective release period, a viable approach is to incorporate a retarding step before the diffusion of the biocide to the surface occurs. A number of such approaches have been introduced. Most are based on reservoir properties of added porous materials such as zeolites and silica (e.g., Edge et al. 2001). Other release-concepts are emerging, addressing release-on-demand (inclusion of nanopackages), slow release, and so-called bioswitches, which have been applied successfully in other areas such as medical applications and food packaging.

Second, most traditional biocides, for example, mercury compounds, are or will soon be under prohibitive rules. In this context, the EU Biocides Directive 98/8/EC (European Parliament and the Council of the EU 1998) reflects a tightened environmental policy. Therefore, European industries are eagerly searching for ecofriendlier alternatives.

### Toward performance requirements and product labeling

The recognition of the crucial role of the interior finish calls for an approved method for assessing the its mold control performance. Such a method is a basic instrument for product labeling and end-user implementation. In addition, control of fungal growth on materials has been identified as a priority in EU member states responding to mandate M/366 (approved November 2004; EU Commission 2005c). The CPD applies to all construction products that are produced for or incorporated within building and civil engineering construction works. It harmonizes all construction products subject to regulatory controls for marking purposes.

Present methods use a single moisture regime and do not explicitly consider effects of transient moisture loads and subsequent material performance in relation to the transient loads. Most tests are based either on a more or less steady-state level of the relative humidity below saturation (Anonymous 1968, 1975, 1978, 1986, 1988a) or unambiguous surface moistening (Anonymous 1988b, 1989a, 1989b). Adan et al. (1999) proposed a new test that considers the effect of indoor climate dynamics.

Pilot application of the test during the past decade yielded a highly reproducible and discriminating picture of material performance in terms of fungal resistance and showed performance that might differ considerably based on the moisture load. Tests were conducted specifically on silicon caulking typically applied in sanitary rooms (Adan and Lurkin 1997a); a wide range of coating types including waterborne interior paints (Adan et al. 1999); specialties such as high-absorbing claddings (Adan and Lurkin 1997b) and ceramic coatings (Sanders 2002a); fiber products, gypsum-based plasters, and wallpapers including glues (Adan et al. 1999); and cement-based panels (Sanders 2002b). Fungal resistance was found to be a product-based feature and application oriented, emphasizing the importance of indoor climate dynamics for mold resistance. These findings laid the foundation for an approved product qualification system in the Netherlands with respect to fungal resistance. Such a system is a step toward performance requirements in building regulations. Moreover, product labeling provides support to end users, i.e., tenants and building owners, the actual occupants.

Labeling is defined by a three-level classification system: I, resistant; II, fairly resistant; and III, sensitive (Table 1). These definitions are based on analysis of the entire growth pattern as a function of time (Adan 1995; Adan et al. 1999).

The basic principle underlying the classification system is the potential of most products to exhibit widely divergent behavior as a function of the moisture load. In the past decade, in about 50% of the tested products, steady-state and transient (i.e., condensation) conditions showed highly differing behavior, underlining the importance of considering both climatic conditions in assessing product performance. Consequently, a labeling system should be connected to a recommended application. The best quality (labeled “I”) in terms of resistance reflects that the majority of mold problems occurs in indoor areas with a distinct vapor production [e.g., bathrooms and kitchens in 60 and 40% of cases in the Netherlands, respectively (Anonymous 1993)]. In all other indoor areas, with a more or less steady-state indoor humidity, risks of surface growth are a consequence of interaction of finishing product, building construction—thermal bridging in particular—and average humidity or ventilation. In these cases, product labeling discriminates between fairly resistant products that can be applied on thermal bridges and sensitive products that should be applied only on inner constructions in dry environments.

## Conclusions

We discussed the issue of how to design a healthy building from three viewpoints. The first approach describes sustainable development, focusing on what should be considered in design and land use. Second, the analysis of how occupants affect their indoor air quality links the everyday use of the building to its design. Third, the overview of recent developments in products and materials and their certification and labeling indicates a trend toward addressing current problems.

Sustainable design rediscovers the social, environmental and technical values of pedestrian, mixed-use communities, using existing infrastructures, including main streets and small-town planning principles, and recapturing indoor–outdoor relationships. Sustainable design introduces benign, nonpolluting materials and assemblies with lower energy requirements and higher durability and recyclability.

Humans have a major role in maintaining the healthy indoor environment, especially in residences. This role includes personal cleaning habits and other personal behaviors. The occupants of the building decide the types of consumer products to be used and furnishings and appliances to be purchased, as well as where and how they are used. Thus, the occupant has a key role in determining the quality of indoor air in his/her residence.

Certification of consumer products and building materials as low-emitting products is a primary control measure for achieving good indoor air quality. Key products in this respect are office furniture, flooring, paints and coatings, adhesives and sealants, wall coverings, wood products, textiles, insulation, and cleaning products. The finishing materials have a key role in moisture retention and mold growth. The goal of product development is to reduce material susceptibility, to establish performance requirements for finishes in building codes and to require consumer-oriented product labeling.

Training professionals in various fields of design, construction, maintenance, and management of the building is necessary in developing healthier environments for living and work. Dissemination of information concerning the healthiness of the indoor environment and what a consumer can do about it is essential to increase root-level activities toward obtaining and maintaining healthier buildings.

## Figures and Tables

**Table 1:** The Dutch classification system for fungal resistance of interior finishes.

Class	Quality	Recommended application
I	Resistant	Indoor environments with transient moisture loads such as bathrooms, kitchens, production processes, swimming pools
II	Fairly resistant	All other indoor areas, with a more or less steady-state indoor humidity, such as living rooms, attics, storage rooms, or depots
III	Sensitive	Only on inner constructions not being part of the building envelope in environments other than class I

Adapted from Adan et al. (1999).
